# Split dose versus single bolus gadolinium administration in ecv calculation at 3 tesla cmr

**DOI:** 10.1186/1532-429X-17-S1-P257

**Published:** 2015-02-03

**Authors:** Adam K McDiarmid, David M Higgins, David A Broadbent, David P Ripley, Peter P Swoboda, Ananth Kidambi, Bara Erhayiem, Tarique A Musa, Laura E Dobson, Pankaj Garg, John P Greenwood, Sven Plein

**Affiliations:** Philips Centre, Philips Healthcare, Guilford, UK; Multidisciplinary Cardiovascular Research Centre & Leeds Institute of Cardiovascular and Metabolic Medicine, University of Leeds, Leeds, UK

## Background

Diffuse myocardial fibrosis may be quantified with cardiovascular magnetic resonance (CMR) by calculating extra-cellular volume (ECV) from native and post-contrast T1 values. Previous studies have used either infusion or single bolus contrast administration. In clinical practice however split dose contrast injection is used as part of a stress/rest protocol in stress perfusion studies. The effects of using such an injection regime on ECV calculation is unknown.

This study aimed to assess the effects of split dose versus single bolus contrast administration on ECV calculation.

## Methods

Ten healthy volunteers were studied on a 3.0 Tesla (Philips Achieva TX) MR system and underwent three separate CMR studies over a mean of 30 days. In one study, contrast was administered as a single bolus (Gadovist 0.15mmol/kg). In two further CMR studies, contrast was given in two boluses (0.075mmol/kg per bolus) as part of an adenosine stress/rest perfusion protocol, separated by 12 minutes. T1 maps were acquired pre contrast and 15 minutes following the single bolus or second contrast injection. T1 measurements were made in the inter-ventricular septum. Means and standard deviations were compared between MOLLI T1 estimates and ECV calculated.

## Results

**Figure 1:**
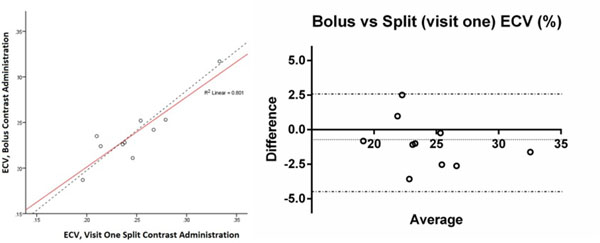
Bland Altman plot of agreement between ECV estimated using single bolus and split-dose contrast administrations (bias -0.993, 95% CI -4.495 to 2.509, r^2^=0.801, p=0.00).

**Figure 2:**
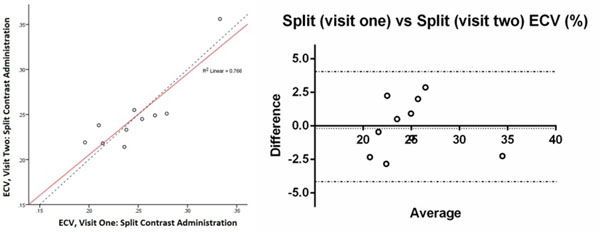
Bland Altman plot of agreement of ECV estimates between visit 1 and 2 using split-dose administration (bias -0.018, 95% CI -4.045 to 4.009, r^2^=0.766, p=0.00)

Volunteer mean age was 27 ± 3yrs, BSA corrected LVEDV (101 ± 12ml/m2) and LV mass (52 ± 7 g/m2) were normal. No perfusion defects or scar were identified in the 10 volunteers. ECV agreed between bolus and split dose contrast administration (coefficient of variability 5.78%, bias -0.993, 95% CI -4.495 to 2.509, r^2^=0.801, p>0.001)(figure 1). Inter-study agreement with split dose administration was good (coefficient of variability, 5.67%, bias -0.018, 95% CI -4.045 to 4.009, r^2^=0.766, p>0.001)(figure 2).

## Conclusions

ECV quantification using split dose contrast administration is reproducible and in healthy controls agrees well with previously validated methods. This suggests that perfusion CMR studies may incorporate assessment of tissue composition by ECV based on T1 mapping.

## Funding

AKM is funded by a British Heart Foundation Project Grant (PG/14/10/30641)

DAB has a Research Doctoral Fellowship from the National Institute for Health and Research (DRF-2012-05-155)

SP is funded by a British Heart Foundation Senior Fellowship (FS/10/62/28409).

